# Chaetognatha of the Namibian Upwelling Region: Taxonomy, Distribution and Trophic Position

**DOI:** 10.1371/journal.pone.0053839

**Published:** 2013-01-16

**Authors:** Karolina Bohata, Rolf Koppelmann

**Affiliations:** Institute for Hydrobiology and Fisheries Science, Center for Earth Systems Research and Sustainability, University of Hamburg, Hamburg, Germany; Institute of Marine Research, Norway

## Abstract

In October 2010, the vertical distribution, biodiversity and maturity stages of Chaetognatha species were investigated at four stations located off Walvis Bay, Namibia. Seventeen species were detected and classified as pelagic, shallow-mesopelagic, deep-mesopelagic and bathypelagic species based upon the weighted mean depth derived from their average vertical distribution. High abundances of Chaetognatha were found in the upper 100 m at all stations of the Walvis Bay transect with a maximum value of 20837 ind. 1000 m^−3^ at the outer shelf station near the surface. The community was dominated by species of the Serratodentata group. Furthermore, the distribution of Chaetognatha did not seem to be influenced by low oxygen concentrations. Stable isotope ratios of carbon and nitrogen in Chaetognatha were determined for seven different areas located off northern Namibia. The values of δ^15^N ranged from 6.05 ‰ to 11.39 ‰, while the δ^13^C values varied between −23.89 ‰ and −17.03 ‰. The highest values for δ^15^N were observed at the Walvis Bay shelf break station. The lowest δ^13^C values were found at the Rocky Point offshore station, which was statistically different from all other areas. Stable isotopes of carbon and nitrogen were determined for four taxa (*Sagitta minima,* Planctonis group, *Sagitta enflata, Sagitta decipiens*). In this case, the δ^15^N values ranged from 6.17 ‰ to 10.38 ‰, whereas the δ^13^C values varied from −22.70 ‰ to −21.56 ‰. The lowest δ^15^N values were found for *S. minima*. The C- and N-content revealed maximum C-values for *S. decipiens* and maximum N-values for the Planctonis group. The C:N ratio of Chaetognatha ranged between 5.25 and 6.20. Overall, Chaetognatha are a diverse group in the pelagic food web of the Benguela Upwelling System and act as competitors of fish larvae and jelly fish by preying on copepods.

## Introduction

Chaetognatha are transparent marine metazoans which live in all marine habitats and are an important group of carnivorous zooplankton in pelagic food webs [1]. They are active predators grasping prey with rigid hooks [2]. The typical and numerically dominant prey of Chaetognatha are copepods, but larger organisms such as fish larvae [3], polychaets [4] or euphausiids are also captured. Chaetognatha even prey on their own kind. The feeding behaviour of Chaetognatha is influenced by prey abundance and predator size [3].

Chaetognatha can be found throughout the whole water column from the surface to several thousands meters depth. The main parameters influencing the vertical distribution are temperature, age of specimen and light intensity [2]. Marazzo & Nogueira [5] suggest that prey abundances could play an important role for the spatial and temporal distribution of Chaetognatha. Other studies assume that low oxygen contents reduce the abundance of Chaetognatha as well [4]–[6], [7]. Many Chaetognatha undergo ontogenetic migration [8] by moving to deeper layers for spawning [9]. Moreover, some species are related to specific environmental conditions, so they can act as indicators of different water masses [2].

The Benguela Upwelling System (BUS) is one of the major eastern boundary upwelling systems of the world [10]. It is situated off the west coast of Africa between 15–37°S and 0–20°E. The BUS spans from Cape of Good Hope in the south along the southern coast of South Africa into Angolan waters [11] and is influenced by along-shore winds [12]. The southern Benguela system is affected by oceanographic processes from the South Atlantic and the Indo-Pacific Oceans. Warm surface water moves from the Indo-Pacific into the Atlantic Ocean mostly in ring-formations which are shed from the reflection of the Agulhas current [11]. The northern Benguela system is affected by the Angola-Benguela Front, resulting in seasonal and inter-annual changes of the intensity of upwelling cells at Cape Frio and Lüderitz [10]. Main characteristics of the BUS are extensive frequently forming oxygen minimum layers (OML) [13]. A shallow OML is located on the shelf between 50 m and 150 m depth and a deeper one at the shelf break in about 300 m depth [14]. The OML of the central BUS is influenced by complex interactions between the remotely forced shelf boundary conditions, seasonal thermocline variability and biogeochemical carbon fluxes [15].

Several authors recently reported on the distribution, feeding and diel migration of Chaetognatha species in the Benguela Upwelling Region [16], [17]–[18]. However, only Duró and Gili [17] described the influence different water masses in the upwelling area have on the horizontal and vertical distribution of Chaetognatha along an onshore-offshore transect off Walvis Bay, Namibia. Some studies were done on the trophic position of Chaetognatha in the food web, but little is known about this topic concerning the BUS. The aim of this study was therefore (1) to asses the distribution and biodiversity of Chaetognatha depending on the water masses and especially to analyse the effect oxygen minimum layers have on the vertical distribution; (2) to assess the differences in vertical and horizontal distribution of different developmental stages of Chaetognatha species; and (3) to asses the isotopic signatures of different Chaetognatha taxa and different areas in the BUS. In upwelling systems, the phytoplankton and zooplankton communities are transported offshore towards the frontal area. There, due to different water masses, planktonic organisms become highly concentrated and thus attract small pelagic predators and other planktivorous nekton [19]. Therefore, we assume that the abundance of Chaetognatha increases in this zone as well. However, the diversity should be highest at the offshore site, where typically a more diverse oceanic food web exists [20].

## Materials and Methods

### Sample Collection

**Figure 1:**
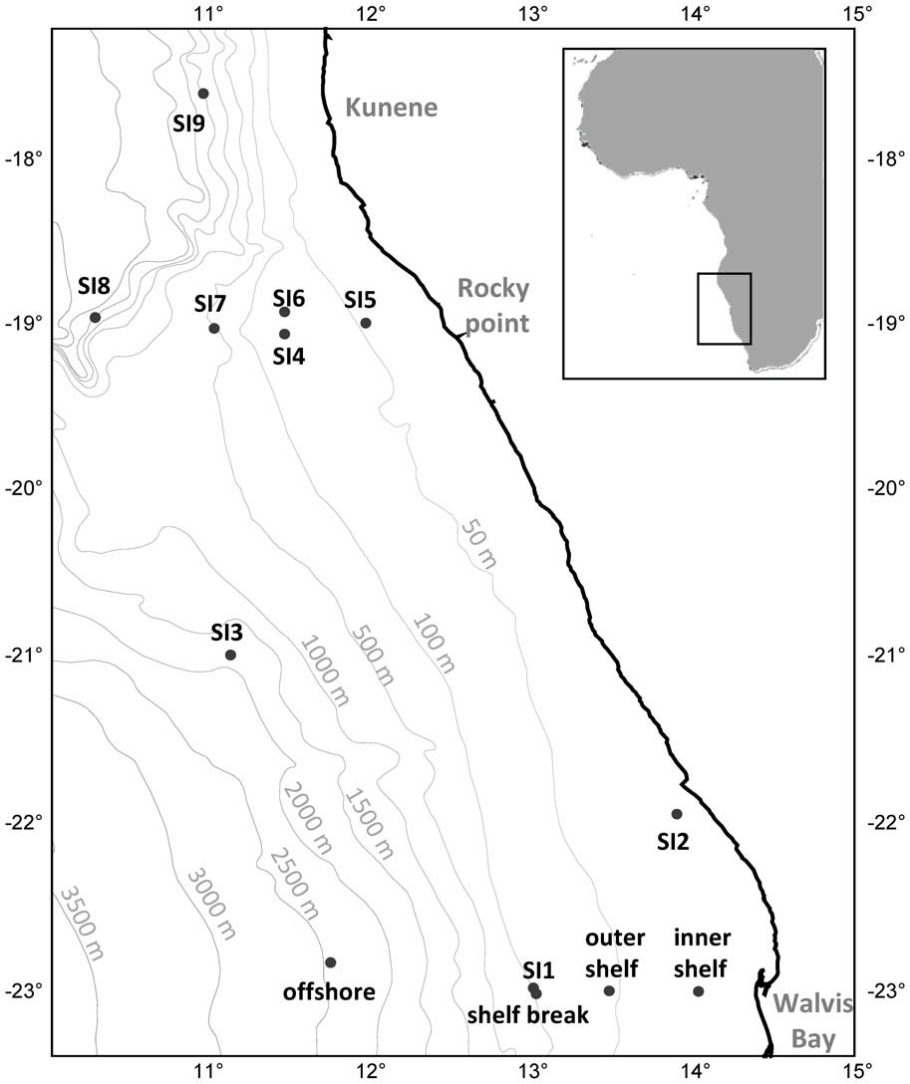
Research area with and sampled stations. SI = stable isototpe samples; WB = samples for taxonomy and distribution.

Zooplankton samples were taken on the British research vessel Discovery, cruise 356, at several locations off the Namibian coast in September and October 2010 (Figure 1). The samples for the determination of the Chaetognatha distribution were collected at 4 stations on the Walvis Bay transect (Figure 1, Table 1). Stations were located on the inner and outer shelf as well as the shelf break and offshore. The samples were taken with a double 1 m^2^-MOCNESS (Multiple Opening and Closing Net and Environmental Sensing System, [21]). A MOCNESS consists of 18 nets of 333 µm mesh size, which can opened and closed sequentially at different water depths. The towing speed was 2 knots at a heaving speed of 0.5 m·s^−2^. Furthermore, the double 1 m^2^-MOCNESS was equipped with a flow meter. The volume filtered through each net was determined by the MOCNESS program, taking the towing angle into account. Temperature, salinity and oxygen related to the depth of the water column were measured with a CTD (Seabird 911+) at all sampled stations.

**Table 1:** Sampling data for the analyses of the vertical and horizontal distribution of Chaetognatha species on the Walvis Bay transect.

Station	Date	UTC	Latitude	Longitude	Waterdepth [m]	Haul intervals [m] and (# of samples)
inner shelf	22.09.2011	21∶43 (n)	23°00′S	14°03′N	132	0–25–50–100 (3)
outer shelf	22.09.2011	00∶02 (n)	23°00′S	13°30′N	231	0–25–50–100–150 (4)
shelf break	19.09.2011	22∶20 (n)	23°01′S	13°03′N	437	0–25–50–100–150–200–250–300–350 (8)
offshore	20.09.2011	17∶16 (n)	22°52′S	11°47′N	2998	0–25–50–100–200–400–600–800–1000–1250–1500–1750–2000–2250–2500–2620 (15)

Local time = UTC +2 h; n = night, d = day.

After rinsing the nets with filtered seawater, the zooplankton material was concentrated and fixed in a 4% formaldehyde-seawater solution buffered with sodium-tetraborate.

Samples for the determination of stable isotopes were taken by opportunity from different nets used by other working groups (double 1 m^2^-MOCNESS, WP-2, Ring Trawl, Driftnet) at 9 stations (SI1– SI9) located on the shelf, shelf break and offshore north of Walvis Bay (Figure 1; Tables 2, 3). The WP-2 net had a mesh size of 300 µm and was towed vertically from 50 m depth to the surface. The Ring Trawl had a mesh size of 1000 µm and was towed horizontally over the stern. The driftnet, mesh size 30 µm, was released into the water and then transported away by the current. The samples were rinsed in fresh water and deep frozen at −80°C.

**Table 2:** Sampling data for stable isotope analyses of areas.

Station	Date	UTC	Latitude	Longitude	Si	Water depth [m]	Net	Sampling depth [m]
SI1	08.10.2011	08∶47 (d)	22°59'S	13°02'E	5	391	MOCNESS	0–300
SI2	24.09.2011	16∶40 (d)	21°57'S	13°55'E	4	56	WP-2	0–50
SI3	27.09.2011	07∶20 (d)	21°00'S	11°10 'E	9	2151	Driftnet	0–2
SI4	30.09.2011	01∶02 (n)	19°04'S	11°30'E	11	200	MOCNESS	25–50
SI5	29.09.2011	12∶04 (d)	19°00'S	12°00'E	2	198	MOCNESS	0–100
SI6	29.09.2011	00∶26 (n)	18°56'S	11°30'E	8	285	MOCNESS	0–250
SI7	28.09.2011	22∶30 (n)	19°02'S	11°04'E	13	1050	Ring Trawl	0–50
SI8	02.10.2011	14∶20 (d)	18°58'S	10°20'E	16	3173	MOCNESS	25–50
SI9	01.10.2011	23∶15 (n)	17°36'S	11°00'E	1	112	Ring Trawl	0–50

Local time = UTC +2 h; n = night, d = day, Si = number of subsamples.

**Table 3:** Sampling data for stable isotope analyses of species.

Species	Station	Date	UTC	Latitude	Longitude	Si	Water depth [m]	Net
*Sagitta minima*	SI5	29.09.2011	12∶04 (d)	19°00'S	12°00'E	6	198	MOCNESS
*Sagitta enflata*	SI2	24.09.2011	16∶40 (d)	21°57'S	13°55'E	5	56	WP-2
*S. enflata*	SI3	27.09.2011	07∶20 (d)	21°00'S	11°10 'E	2	2151	Driftnet
*S. enflata*	SI9	01.10.2011	23∶15 (n)	17°36'S	11°00'E	4	112	Ring Trawl
*Sagitta decipiens*	SI3	27.09.2011	07∶20 (d)	21°00'S	11°10 'E	3	2151	Driftnet
Planctonis group	SI3	27.09.2011	07∶20 (d)	21°00'S	11°10 'E	3	2151	Driftnet

Local time = UTC +2 h; n = night, d = day, Si = number of subsamples.

### Sample Processing

#### Distribution and abundance

The 1 m^2^-MOCNESS samples were weighed and transferred into a sorting fluid composed of 94.5% fresh water, 5.0% propylene glycol and 0.5% propylene-phenoxetol for the analysis of taxonomic composition and distribution. The samples were separated into different fractions for further analysis using a set of sieves. Chaetognatha species were extracted from these subsamples. All Chaetognatha species were classified into three maturity stages following Zo [22]. Stage I: young individuals without visible ovaries; Stage II: individuals with visible ovaries with immature ova that varied in size and with well developed gonads; Stage III: individuals with some mature ova in the ovaries. The species were counted and standardized to number of individuals 1000 m^−3^.

To determine the mean vertical distribution of the species, the weighted mean depth (WMD) was calculated according to Perry *et al.* [23]:
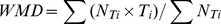
where *N_Ti_* is the abundance in the depth layer *i* and *T_i_* the mean depth of the sampling interval in meter. Individual components of diversity were calculated by different indices. Richness was represented by the number of species per station. The diversity index (H) was calculated as follows [24]:



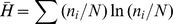
where N is the total abundance of all species, n_i_ represents the abundance of the *i* species.

To examine how the abundances of species differ in a community, the evenness (J) at each station was calculated using Pilou’s [25] formula with diversity index (H):
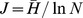



The dominance index was calculated according to Simpson [26]:
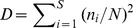
where S is the total number of species at the station, n*_i_* is the number of individuals of the *i* species and N the total number of individuals of the Chaetognatha community.

#### Stable isotopes

A stable isotope analysis is a reliable method to characterize the structure of the food web and to investigate the nutrient dynamics as well as pathways of energy flow within ecosystems [27]–[28]. On the vessel, Chaetognatha taxa were extracted from the subsamples, measured and frozen at –20°C. In the laboratory, all samples were defrosted and weighed wet with an analytical balance of an accuracy of 0.1 mg, then dried in a freeze-dryer at –40°C for 24 h. The dried samples were pulverised using a porous ceramic mortar and pistil. Afterwards, the dry weight was determined.

The carbon and nitrogen content as well as stable isotopes values of the different samples were obtained using a Thermo Finnigan Delta V Isotope ratio mass spectrometer (EA-1112 CHN-Analyser) at the stable isotope laboratory of the Museum für Naturkunde - Leibniz-Institut für Evolutions- und Biodiversitätsforschung of the Humboldt-Universität in Berlin. Results are related to standard atmospheric nitrogen or Peedee Belemnite and are expressed as deviations from the standard in parts per thousand as follows:
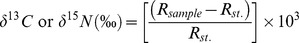
where R_st._ is the standard (^13^C/^12^C) or (^15^N/^14^N).

Nitrogen isotopic compositions reflect dietary relationships within the food web [29]. Nitrogen isotope ratios become enriched by 3–4 ‰ per trophic level [30], [31]–[32]. Stable carbon isotope ratios are useful for identifying the source of the diet [32] due to the fact that the enrichment between successive trophic levels is relatively small, about 0.5–1.0 ‰ [29]–[32].

The stable nitrogen and carbon data of four abundant and easily detectable taxa (*S. enflata, S. minima,* Planctonis group, *S. decipiens*) as well as data from different stations (SI1– SI9) were statistically analysed using one-way ANOVA with Tukey-HSD Post-Hoc-Test. In all cases, a 95% significance level was assumed.

Additionally, following variables were calculated: the relative amount of dry weight (DW) as a function of wet weight (WW), dry weight and wet weight per individual, relative amount of carbon and nitrogen as well as the C:N ration. Data were tested statistically using the Kruskal-Wallis-test for general differences, followed by the Mann-Whitney-U-test for specific differences.

## Results

### Oceanography

**Figure 2:**
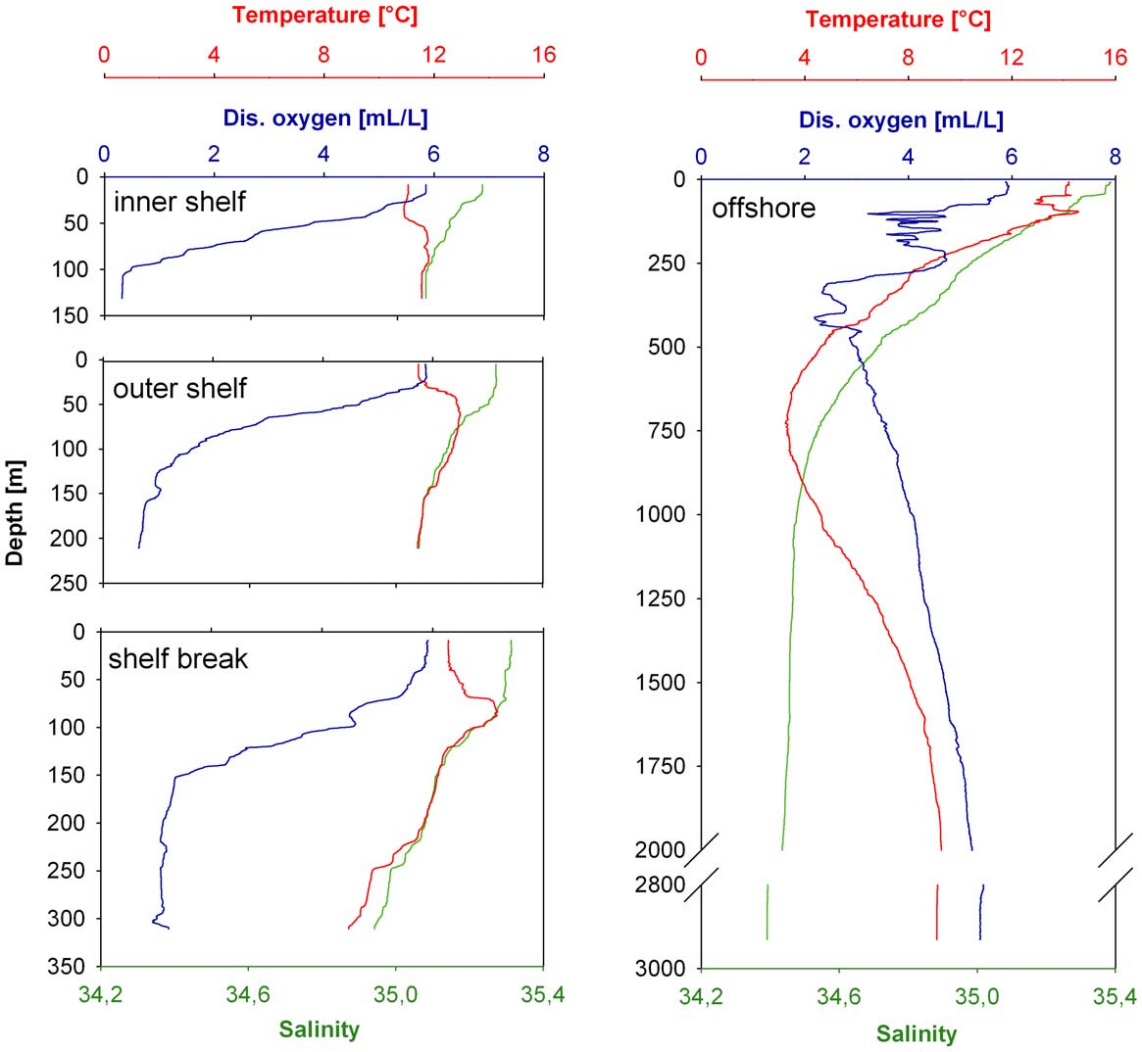
Temperature, salinity and oxygen values of the four stations located on the Walvis Bay transect. A, inner shelf station. B, outer shelf station. C, shelf break station. D, offshore station.

The oceanographic data revealed a typical upwelling situation in the Central Benguela Region with lower temperatures around 14°C at the surface and low oxygen concentrations near the bottom. Oxygen is exhausted during bacterial decomposition of the large amounts of sunken organic material from the productive surface layer (Figure 2).

On the inner shelf (133 m water depth; Table 1), the temperature decreased slightly from ∼15.0°C near the surface (0–25 m) to ∼11.0°C at 132 m. The salinity was almost constant with depth (∼35.1). Near the bottom, oxygen concentrations were at a very low level, but above zero. The oxygen concentration in the surface layer reached almost 6 mL·L^−1^ (Figure 2A).

The outer shelf station (231 m water depth; Table 1) was characterised by temperatures of ∼14°C in the surface layer decreasing slightly to ∼12°C at 200 m. The salinity and dissolved oxygen patterns are comparable to those of the inner shelf station. The salinity was more or less stable with depth (∼35.2), while the dissolved oxygen values reached a relative maximum in the surface layer (∼6 mL·L^−1^). There was a sharp decrease of dissolved oxygen values between 50 m and 150 m, whereas the oxygen concentrations were less than 1.0 mL·L^−1^ between 150 m and 200 m (Figure 2B).

At the shelf break station (437 m water depth; Table 1), the temperature was slightly higher than 14°C near the surface and decreased to below 12°C near the bottom. The salinity maximum was located at a depth of around 75 m (∼35.3) and decreased to ∼34.8 near the bottom. High dissolved oxygen concentrations (∼ 5.9 mL·L^−1^) were found in the surface layer. Below 150 m, a dissolved oxygen minimum of around 1.0 mL·L^−1^ was discovered (Figure 2C).

At the offshore station (2998 m water depth; Table 1), a thermocline was detected at 50 m depth. The surface temperature was around 16.0°C and decreased continuously to 4.0°C at ∼1000 m. The salinity ranged from 35.2 to 35.3 in the upper 100 m and decreased with increasing depth to 34.4 at 750 m. Between 750 m and 2000 m the values increased once more to 34.9. The maximum oxygen concentration (∼8.0 mL·L^−1^) was found in the surface layer. The lowest dissolved oxygen value (∼2.0 mL·L^−1^) was determined at a depth of 400 m. Below 2000 m depth, the temperature, salinity and oxygen were almost constant with values of ∼2.0°C, 34.9 and 5.4 mL·L^−1^ (Figure 2D).

### Abundance and Distribution

#### Abundance

**Figure 3:**
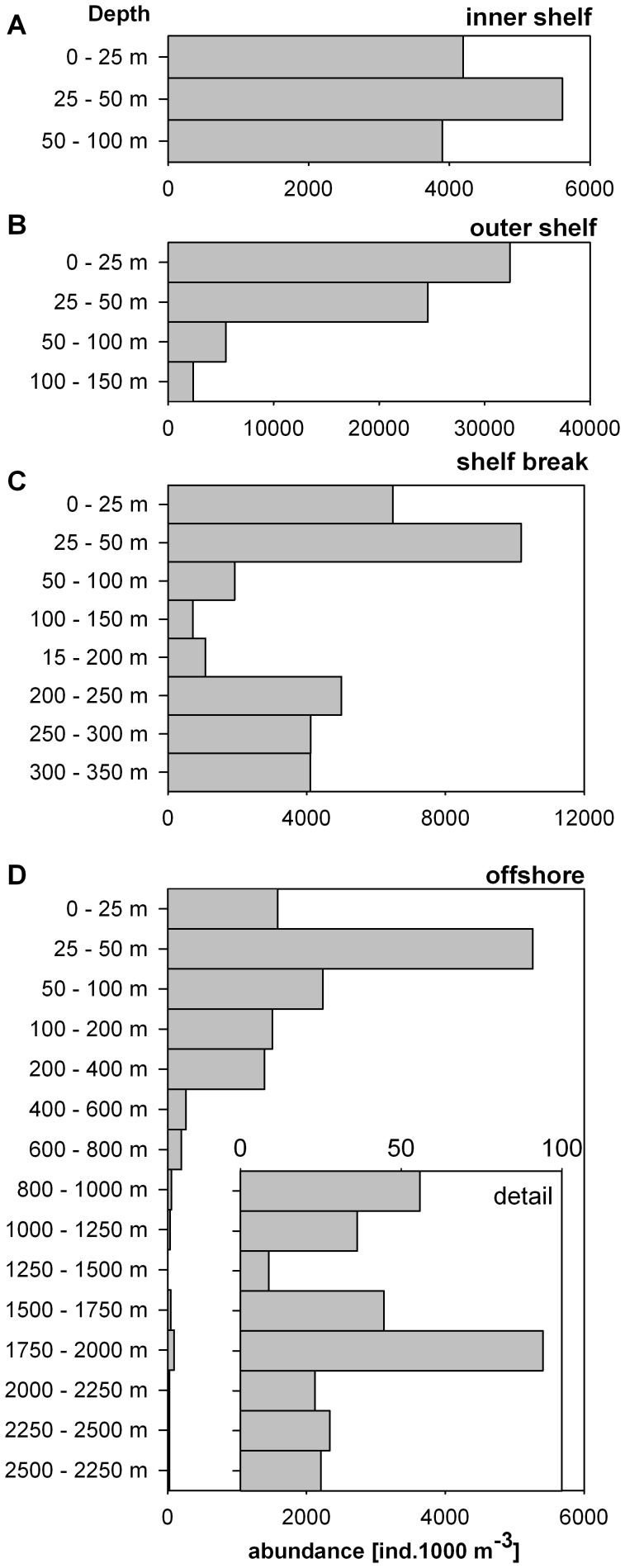
Abundance and vertical distribution of Chaetognatha on the Walsvis Bay transect. A, inner shelf station. B, outer shelf station. C, shelf break station. D, offshore station. Note the different scales.

At the inner shelf station, the maximum abundance of Chaetognatha was detected between 25 m and 50 m (5606 ind. 1000 m^−3^). The abundance in the surface layer (0–25 m) and at 50 m to 100 m reached values of 4194 ind. 1000 m^−3^ and 3902 ind. 1000 m^−3^, respectively (Figure 3A).

The highest abundance of Chaetognatha on the Walvis Bay transect was found in the surface layer at the outer shelf station (32403 ind. 1000 m^−3^). The abundance decreased with depth to 2376 ind. 1000 m^−3^ from 100 m to 150 m (Figure 3B).

The shelf break station showed a similar pattern as the inner shelf station. However, with lower densities of 1017 ind. 1000 m^−3 ^in the 25 m to 50 m layer (Figure 3C), while abundance at the surface layer (0–25 m) reached 6485 ind. 1000 m^−3^. From 50 m to 150 m a decrease of Chaetognatha abundance was found with a minimum value at 100 m to 150 m depth of 724 ind. 1000 m^−3^. Between 200 m and 350 m, the abundances were more or less constant with values below 5000 ind. 1000 m^−3^.

Relatively low concentrations of Chaetognatha were observed at the offshore station. The maximum abundance was found in the layer between 25 and 50 m depth (5232 ind. 1000 m^−3^). Between 50 m and 400 m depth, the abundance was relatively constant with values up to ∼ 1400 ind. 1000 m^−3^. Below 400 m depth, the abundances decreased rapidly and reached a minimum of 9 ind. 1000 m^−3^ between 1250 m to 1500 m.

#### Species composition and ontogenetic distribution

The Chaetognatha community on the Walvis Bay transect included seventeen species. The species were assigned to seven groups and one ungrouped species following Casanova [2] (Table 4). Not all three maturity stages were always found within the species. Adults of *Sagitta macrocephala, Sagitta lyra, Sagitta enflata, Sagitta sibogae, Sagitta planctonis* and *Sagitta zetesiois* were not detected. It was not possible to differentiate juveniles from the species of the Serratodentata group due to the fact that the main diagnostic features for identifying stage I and II were not conspicuously discernible in the investigated material. Adults of *Sagitta tasmanica* and *Sagitta serratodentata* were identified on the basis of the gonad and ovary development as well as the shape of the fins. Hence, stages I and II were pooled in the results as Serratodentata group (see Table 5). At all stations, only adults of *S. tasmanica* and *S. serratodentata* were found, so it is assumed that the juveniles identified belong to these two species.

**Table 4:** Chaetognatha groups and species detected on the Walvis Bay transect.

Family	Group	Species
Pterosagittidae	Pterosagitta	*Pterosagitta draco* (Krohn, 1853)
Eukrohniidae	Hamata	*Eukrohina flaccicoeca* (Casanova, 1986)
		*Eukrohina hamata* (Möbius, 1875)
	Fowleri	*Eukrohina bathyantarctica* (David, 1958)
		*Eukrohina fowleri* (Ritter-Záhony, 1909)
Sagittidae	Lyra	*Sagitta lyra* (Krohn, 1853)
		*Sagitta maxima* (Conant, 1896)
	Serratodentata	*Sagitta serratodentata* (Tokioka, 1940)
		*Sagitta tasmanica* (Thomson, 1947)
	Hexaptera	*Sagitta enflata* (Grassi, 1881)
		*Sagitta hexaptera* (d'Orbigny, 1843)
	Minima	*Sagitta decipiens* (Fowler, 1905)
		*Sagitta minima* (Grassi, 1881)
		*Sagitta sibogae* (Fowler, 1906)
	Planctonis	*Sagitta zetesiois* (Fowler, 1905)
		*Sagitta planctonis* (Steinhaus, 1896)
	ungrouped	*Sagitta macrocephala* (Fowler, 1905)

Classification according to Casanova [2].

**Table 5:** Weighted mean depths (WMD) in meter of Chaetognatha species and their maturity stages on the Walvis Bay transect.

	inner shelf			outer shelf			shelf break			offshore		
Species/stages	I	II	III	I	II	III	I	II	III	I	II	III
*P. draco*							37	75	75	42	43	59
*E. flaccicoeca*												1445
*E. hamata*	75			117			267			344	700	
*E. bathyantarctica*												2020
*E. fowleri*												880
*S. lyra*							176			70	43	355
*S. maxima*							287			418	307	356
Serratodentata gr.	34	34		48	30		103	71		94	37	
*S. tasmanica*			19			31						
*S. serratodentata*						19			31			46
*S. enflata*										105		
*S. hexaptera*							199			67	46	87
*S. decipiens*	38	14	15	65	37		214	252	253	92	85	75
*S. minima*				12	19	41	34	42	47	36	72	46
*S. sibogae*	58	73										
*S. zetesiois*							320	325		673	988	
*S. planctonis*							29			69		
*S macrocephala*										1240		

**Figure 4:**
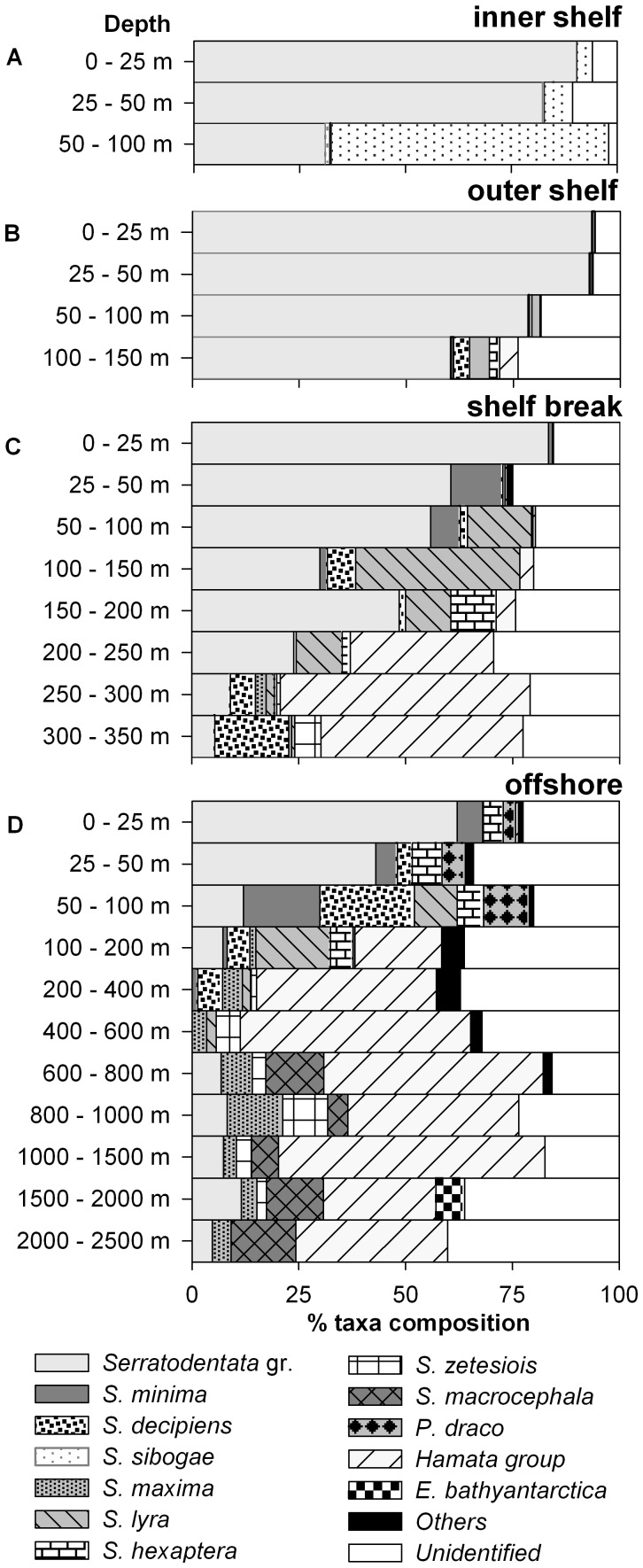
Relative abundance of the Chaetognatha species at the four investigated stations on the Walvis Bay transect. A, inner shelf station. B, outer shelf station. C, shelf break station. D, offshore station. Serratodentata group = stages I and II of Serratodentata group and adults of *S. serratodentata* and *S. tasmanica*. Hamata group = *E. hamata, E. flaccicoeca*. Others = *E. fowleri, S. enflata* and *S. planctonis*.

At the inner shelf station, only three Chaetognatha groups were found: the Serratodentata group, the Minima group and the Hamata group (Figure 4). Five Chaetognatha groups were observed at the outer shelf station: the Serratodentata group, the Hamata group, the Minima group, the Hexaptera group and the Lyra group. Two more groups were identified at the shelf break and offshore stations: the Planctonis group and the Pterosagitta group, which consisted of only one species *Pterosagitta draco*.

Based on the weighted mean (WMD) depths (Table 5), the 17 species identified were classified as pelagic, shallow- or deep-mesopelagic or bathypelagic species (Table 6). Seven of them were characterized as epipelagic species (WMD in the upper 200 m), three species as epipelagic to shallow mesopelagic species (upper 600 m), one species as shallow-mesopelagic (200–600 m), two species as shallow- to deep-mesopelagic species (200–1000 m) and four species were attributed to the bathypelagic species (>1000 m).

**Table 6:** The occurrence of the main species in different depth-zones of the study area.

	Epipelagic 0–200 m	Shallow-mesopelagic200–600 m	Deep-mesopelagic600–1000 m	Bathypelagic >1000 m
*P. draco*	X			
*S. tasmanica*	X			
*S. serratodentata*	X			
*S. enflata*	X			
*S. minima*	X			
*S. sibogae*	X			
*S. planctonis*	X			
*S. hexaptera*	X	X		
*S. lyra*	X	X		
*S. decipiens*	X	X		
*S. maxima*		X		
*E. hamata*		X	X	
*S. zetesiois*		X	X	
*S macrocephala*				X
*E. flaccicoeca*				X
*E. fowleri*				X
*E. bathyantarctica*				X

**Figure 5:**
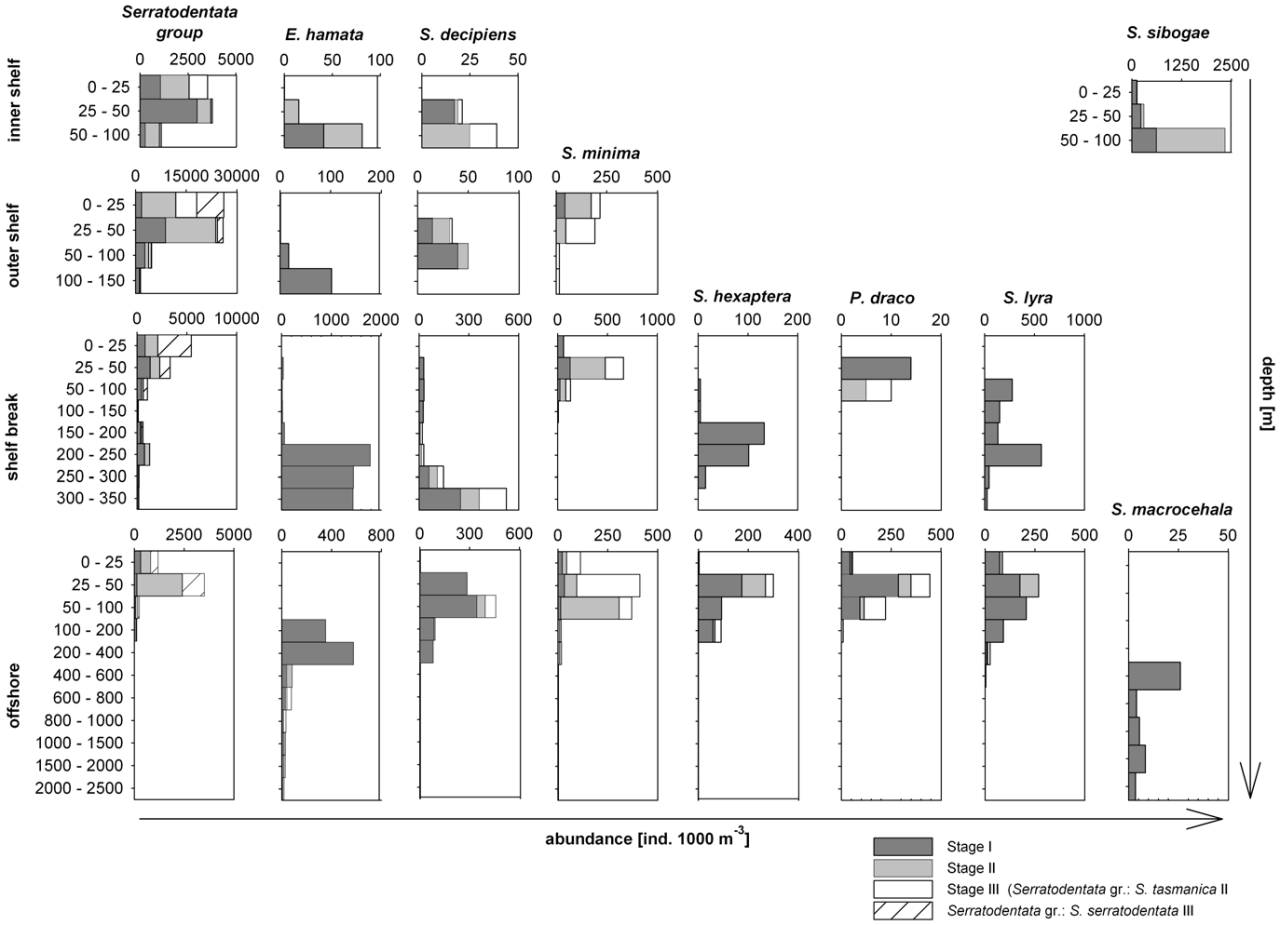
Abundance and vertical distribution of the most numerous species of Chaetognatha and their life stages at all four station. Note the different scales.

**Figure 6:**
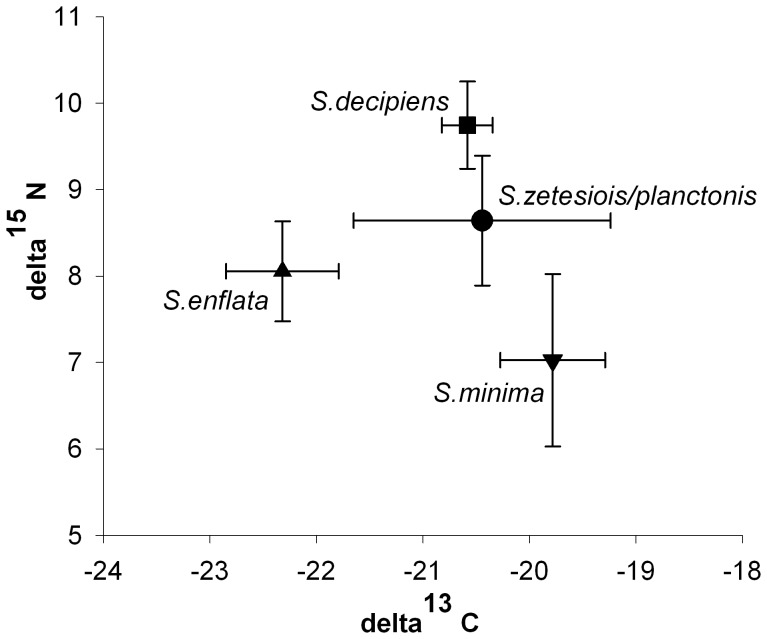
Relationship between δ^13^C and δ^15^N for various areas of northern Namibia. Mean values and standard deviations are shown.

The Serratodentata group, dominating all four stations, belonged to the epipelagic and shallow-mesopelagic community. It constituted 60% of the standing stock at the inner shelf station, 89% at the outer shelf station, 36% at the shelf break station and 40% at the offshore station (Figure 4). In the surface layer (0–50 m depth), the Serratodentata group accounted for over 80% of the standing stock at the inner and outer shelf station, over 60% at the shelf break station and over 43% at the offshore station (Figure 5). The highest concentration of this group was found at the outer shelf station accounting for 25118 ind. 1000 m^−3^ at the surface layer (0–25 m) and 18282 ind. 1000 m^−3^ at the depth from 25 m to 50 m (Figure 6). The distribution pattern for the stages I and II showed similarities at all four stations with high abundances between 25 m and 50 m. The adults of *S. tasmanica* and *S. serratodentata* preferred the upper layers between 0 m and 50 m.

Dominant epipelagic and shallow-mesopelagic species were *Sagitta minima, Sagitta decipiens* and *Sagitta sibogae* from the Minima group. The Minima group was the second most dominant group at the inner shelf station, compromising 20% of the standing stock (Figure 4). The proportion of the Minima group was relatively high at the shelf break station as well as at the offshore station, constituting 5% and 11% of the standing stock, respectively. However, only *S. decipiens* was found at all stations. The abundance of this species was high at the shelf break and offshore stations. Yet, the developmental stage I of *S. sibogae* occurred exclusively at the inner shelf station between 50 m to 100 m depth. *S. minima* preferred the upper layers of the shelf break and offshore stations between 0 m to 150 m depth (Figures 5 and 6) with peak abundances of 172 ind. 1000 m^−3^ in the upper 25 m at the outer shelf station, 661 ind. 1000 m^−3^ at the shelf break station between 25 m and 50 m and 412 ind. 1000 m^−3^ at the offshore station at the same depth range.

The Hamata group was found at all four stations on the Walvis Bay transect. The relative densities at the inner and outer shelf stations were low (Figure 4). This group contributed with less than 1% to the standing stock at the inner and outer shelf stations. Relative amounts of 14% and 10% were recorded at the shelf break and offshore stations, respectively. At the inner and outer shelf as well as shelf break stations, only stage I of *Eukrohnia hamata* was found. It preferred layers near the bottom or at depths greater than 200 m with abundances over 1400 ind. 1000 m^−3^ at the shelf break station and 350 ind. 1000 m^−3^ at the offshore station (∼30% of the total at each depth layer). Some stage II of *E. hamata* and adults of *Eukrohnia flaccicoeca* were detected at the offshore station between 600 and 1000 m depth. *E. flaccicoeca* reached a maximum abundance of 33 ind. 1000 m^−3^ in the layer between 600 m and 800 m (Figure 6). Due to the low abundances of species belonging to the Hamata group (*E.hamata, E. flaccicoeca*), these species were pooled in Figures 5 and 6.

Individuals of *P. draco* were mainly found in oceanic waters on the Walvis Bay transect and occurred at depths between 50 m and 200 m. The abundances at the shelf break station were very low (Figure 6). The maximum density of 444 ind. 1000 m^−3^ was found in the depth layer between 25 m to 50 m at the offshore station. The immature stage I dominated the population, it consisted of 141 ind. 1000 m^−3^ in the upper 100 m at the offshore station.

*Sagitta hexaptera* and *Sagitta enflata* belong to the Hexaptera group and were mainly found at the offshore station (Figure 4). Only maturity stage I was found at the shelf break station, occurring in depth layers between 150 m and 300 m, but all three stages were observed at the offshore station (Figure 6). At this station, *S. hexaptera* was found in the upper 200 m with a maximum abundance of 301 ind. 1000 m^−3^ at 25 m to 50 m (Figure 5).

The Lyra group was sampled at the shelf break stations and offshore station (Figure 4). *Sagitta lyra* showed a similar distribution pattern as *S. hexaptera* with stage I occurring more often at the shelf break station and less often at the offshore station. No adults of *S. lyra* were found at the shelf break station. At the offshore station, the density of stage I reached a maximum value of 207 ind. 1000 m^−3^ from 50 m to 100 m. Stage II individuals were observed in the upper layers with a peak abundance of 95 ind.1000 m^−3^ between 25 m and 50 m. *S. maxima* was mainly found at the offshore station between 100 m and 2500 m depth (Figure 6).

The Planctonis group was detected at the shelf break and offshore stations (Figure 4). It consisted of two species, *Sagitta planctonis* and *Sagitta zetesiois*. *S. planctonis* is an epipelagic and shallow-mesopelagic species found sporadically in the upper 400 m. *S. zetesiois* is a deep-mesopelagic to bathypelagic species found in deeper water layers below 250 m at the shelf break station and below 400 m at the offshore station (Figure 5 and 6).

*Sagitta macrocephala* is a deep-mesopelagic and bathypelagic species (Figures 5 and 6). Only stage I was found at the offshore station with a peak density of 26 ind. 1000 m^−3^ between 600 m and 800 m. The abundances in the deeper layers were relatively low with up to 8 ind. 1000 m^−3^ (Figure 6).

#### Diversity, dominance and evenness

The richness and diversity of Chaetognatha species showed a tendency to increase from the shelf towards the open ocean (Table 7). The diversity of the Chaetognatha community (Shannon-Weaver index H) was much lower at the inner shelf station (H = 0.3) compared to the offshore station (H = 1.38). Furthermore, the evenness (J) was also lowest at the inner shelf station (J = 0.21) and highest at the shelf break and offshore station (J >0.50). The values of the Simpson Dominance Index (D) showed a higher dominance of one group (Serratodentata group) at the shelf and shelf break stations (D = 0.31–0.55) compared to the offshore station (D = 0.03).

**Table 7:** Number of species, values of the Shannon–Weaver index H, the Evenness J and the Simpson index D at the four sampling sites.

	innershelf	outershelf	shelfbreak	offshore
Number of taxa	4	7	10	15
Shannon-Weaver index H	0.30	0.63	1.21	1.38
Evenness J	0.21	0.33	0.52	0.51
Simpson index D	0.55	0.31	0.42	0.03

### Stable Isotopes

Carbon and nitrogen stable isotope ratios of Chaetognatha were determined for seven different areas (9 stations SI1– SI9) located on the shelf, at the shelf break and offshore off northern Namibia (Figure 1). All sampled individuals were pooled according to the stations. Additionally, carbon and nitrogen stable isotopes were measured for the following four taxa*: S. minima,* Planctonis group (*S. planctonis* and *S. zetesiois*), *S. enflata, S. decipiens.* For the determination of stable isotopes of the four taxa, samples from different areas were pooled. Due to the fact that it was not possible to differentiate between two species from the Planctonis group (*S.zetesiois* and *S. planctonis)* on the vessel, these two species were pooled (Figure 6).

#### Differences between areas

Significant differences in δ^13^C were found between the areas (One-way ANOVA: F = 4.904; P<0.001; α <0.050). The values for δ^13^C ranged from −23.89 ‰ to −17.03 ‰. The lowest values determined were found at the Rocky Point offshore station (−23.89 ‰ to −21.87 ‰) which were significantly different from all other stations (P<0.050; Table 8). No significant differences were found between the other combinations (P>0.050).

**Table 8:** Summary of One-way ANOVA with Tukey-HSD Post-Hoc-Test investigating differences in δ^13^C and δ^15^N values of different locations in the northern Namibian Upwelling System.

	Kunene (O)	W. Bay (SB)	SI 2 (S)	SI 3 (O)	R. Point (S)	R. Point (SB)	R. Point (O)
Kunene (O)	–	n.s.	n.s.	n.s.	n.s.	n.s.	P = 0.019
W. Bay (SB)	n.s.	–	n.s.	n.s.	n.s.	n.s.	P = 0.019
SI 2 (S)	n.s.	P = 0.046	–	n.s.	n.s.	n.s.	P<0.001
SI 3 (O)	n.s.	P = 0.040	n.s.	–	n.s.	n.s.	P<0.001
R. Point (S)	n.s.	n.s.	n.s.	n.s.	–	n.s.	P = 0.023
R. Point (SB)	n.s.	P<0.001	n.s.	P = 0.040	P = 0.002	–	P = 0.004
R. Point (O)	n.s.	P = 0.036	n.s.	n.s.	P = 0.024	n.s.	–

n.s. = no significant differences (P>0.050). Upper right values present the results for δ^13^C, lower left values present the values for δ^15^N. O = offshore, SB = shelf break, S = shelf.

**Figure 7:**
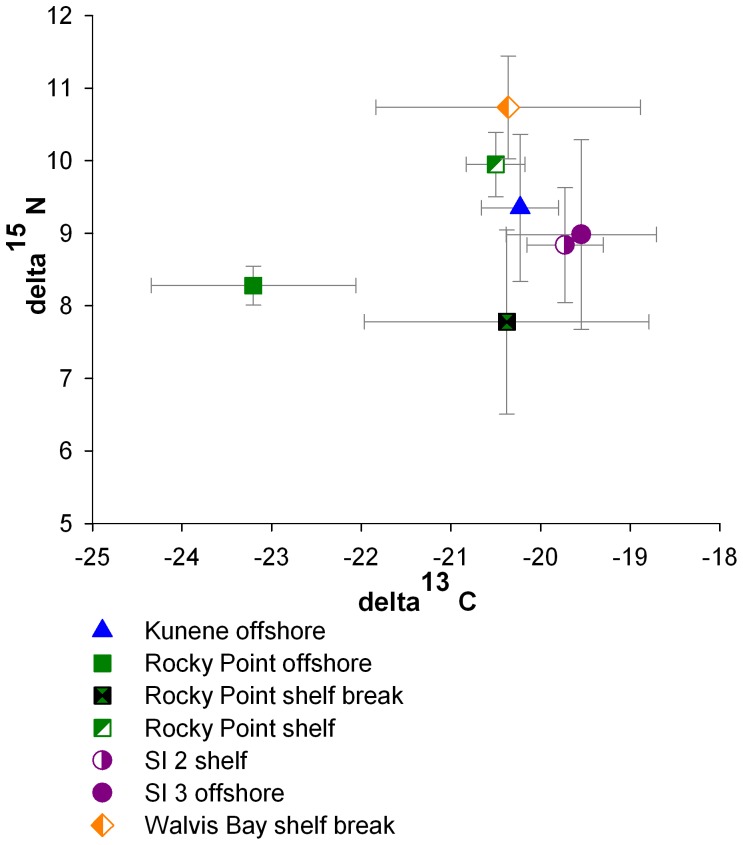
Relationship between δ^13^C and δ^15^N for various species. Mean values and standard deviations are shown.

A one-way ANOVA showed significant differences for δ^15^N values between the areas (one-way ANOVA: F = 7.05; P<0.001; α <0.050). The values varied from 6.05 ‰ to 11.39 ‰ (Figure 7). The highest δ^15^N values were found at the Walvis Bay shelf break station and ranged from 9.93 ‰ to 11.39 ‰. The lowest values for δ^15^N were measured at the Rocky Point shelf break station (6.05 ‰ to 11.07 ‰) which was significantly different from Walvis Bay shelf break, SI2 and Rocky Point offshore station (P<0.001; Table 8). No significant differences in δ^15^N existed between SI2 shelf station, SI3 offshore and Kunene station as well as between Rocky point shelf break and offshore station (P>0.050).

#### Differences between species

Significant differences in δ^13^C were found during comparison of the four taxa (one-way ANOVA: F = 5.63; P = 0.004; α <0.050); the δ^13^C values ranged between −22.70 ‰ and −19.00 ‰. *S. enflata* was statistical different from other three species (P<0.001; Table 9) with δ^13^C values from −22.70 ‰ to −21.56 ‰. The highest δ^13^C values were detected for *S. minima* (−20.29 ‰ to −19.39 ‰) and *S. decipiens* (−20.84 ‰ to −20.37 ‰).

**Table 9:** Summary of One-way ANOVA with Tukey-HSD Post-Hoc-Test investigating differences in δ^13^C and δ^15^N values of different species.

	*S. minima*	*S.enflata*	Planctonis gr.	*S. decipiens*
*S. minima*		P<0.001	n.s.	n.s.
*S.enflata*	n.s.		P<0.001	P<0.001
Planctonis gr.	P = 0.001	n.s.		n.s.
*S. decipiens*	P<0.001	P = 0.038	n.s.	

n.s. = no significant differences (P>0.050). Upper right values present the results for δ^13^C, lower left values present the values for δ^15^N.

A one-way ANOVA showed significant differences for δ^15^N values between the four investigated species (F = 7.05; P<0.001; α <0.050). The values for δ^15^N ranged between 6.17 ‰ and 10.38 ‰ (Figure 6). The δ^15^N values from *S. decipiens* were consistently higher (9.16 ‰ to 10.05 ‰) than those of *S. minima* and *S. enflata* with a significant difference of P<0.001 and P = 0.038, respectively (Table 9). Significant differences were also found between *S. minima* and Planctonis group (P = 0.001). No significant differences in δ^15^N were detected among the other combinations (*S. enflata* and *S. minima*, Planctonis group and *S. enflata*, Planctonis group and *S. decipiens;* P>0.050).

### Carbon and Nitrogen Content

The relative N content of different Chaetognatha species varied from 7.1% to 10.1% of the dry weight (Table 10). The highest value was found in the Planctonis group and the lowest in *S. minima.* The relative C content varied from 32.0% to 51.8%. The highest value was determined for *S. decipiens* and the lowest for *S. minima*. The mean C:N ratio (mol/mol) ranged from 5.25 for *S. minima* to 6.20 for *S. decipiens.* The relative dry weight (DW) varied between 1.9% and 5.2% in *S. enflata* and Planctonis group, respectively. A Kruskal–Wallis-Test showed significant differences in relative C and N contents as well as C:N ratios between the four species (χ^2^ = 10.68, P = 0.014 for C; χ^2^ = 9.28, P = 0.026 for N; χ^2^ = 8.67, P = 0.003 for C:N). The combinations were statistically tested with Mann-Whitney-U-test. Significant differences were detected between *S. minima* and the other species in relative C and N content (P<0.050). *S. minima* was significant different from *S.enflata,* Planctonis group in the C:N ratio (P<0.050). No significant differences were detected between the other combinations (P>0.050).

**Table 10:** The percentage of carbon and nitrogen of different species.

Species	n_s_	n_i_	Size inter. [mm]	WW [mg/ind.]	DW [mg/ind.]	C [% DW]	N [% DW]	C:N
				mean ± SD	mean ± SD	mean ± SD	mean ± SD	mean ± SD
S. minima	6	390	0–10	1.88±0.28	0.06±0.01	32.0±9.6	7.1±1.6	5.25±0.42
*S. enflata*	5	24	0–20	23.3±5.84	0.57±0.31	40.7±8.2	8.8±1.1	5.44±0.44
*S. enflata*	4	8	20–30	95.46±16.31	1.85±0.84	47.4±7.0	9.6±1.1	5.79±0.36
Planctonis gr.	3	18	10–20	49.59±13.28	2.56±1.34	49.9±3.0	10.1±0.1	6.20±0.76
*S. decipiens*	3	180	-	2.26±0.54	0.05±0.02	51.8±4.0	9.8±0.6	5.83±0.31

n_s_ = number of samples, n_i_ = number of individuals, WW = wet weight, DW = dry weight, SD = standard deviation.

## Discussion

Some procedures during sampling and processing of the material made the subsequent identification of Chaetognatha species difficult. Due to destroyed diagnostic features such as fins and head, 17.6% of the species at the inner shelf station, 8.8% at the outer shelf station, 38.6% at the shelf break station, and 29.7% at the offshore station could not be identified (Figure 4). A further obstacle was presented by net sampling with a 333 µm mesh size. This mesh size probably failed to catch small species and juveniles of the large species due to their elongated and slender shapes. Other authors already discussed that plankton nets with 200 µm mesh size could have under-sampled the smaller Chaetognatha species such as *S. minima* [33]. It is well known that the mesh size in plankton nets is a trade-off between the gain in larger species and loss of the smaller ones [34]–[35]. Despite these difficulties, we can present extensive and important data about the vertical and horizontal distribution of the Chaetognatha species with an emphasis on the ontogenetic development as well as being able to determine the trophic position of selected Chaetognatha species in the Benguela Upwelling System (BUS).

### Abundance and Distribution

In general, the vertical distribution of Chaetognatha on the Walvis Bay transect determined in this study is in concordance with results obtained from other studies [17]–[18]. Most of the species (juveniles of the Serratodentata group and S. *tasmanica, S. serratodentata*, *E. hamata, S. decipiens, S. minima, S. hexaptera, P. draco, S. maxima, S. lyra*) were found in the warmer water between 8°C and 16°C in the upper 400 m. The bathypelagic species, except *S. macrocephala*, mainly occurred in depth between 1500 m and 2620 m (the maximum sampling depth at the offshore station), where temperature, oxygen and salinity were almost constant at around 3°C, 5 mL·L^−1^ and 34.9, respectively. Some authors suggested that the distribution [8]–[36], reproduction and mortality rates [37] of some Chaetognatha species depend strictly on specific water masses and are influenced by hydrological factors. In September 2010 (southern spring), small temperature differences in the water column (<3.0°C) were found on the shelf and at the shelf break stations. At the offshore station, a continuous decrease of temperature from 16°C in the surface layer (0–25 m) to 4°C below 750 m was observed. Salinity was relatively stable at the shelf and shelf break stations varying between 34.8 and 35.4. The highest variability in salinity was found at the offshore station (<1.0). Salinity differences as small as those detected in this study probably have no measurable effect on the physiology and distribution of the Chaetognatha [37]–[38]. Some authors [4], [39]–[40] assume that low oxygen contents may reduce the abundance of several species. In this study, an explicit impact of the low oxygen concentrations in the BUS on the Chaetognatha distribution was not detected. Low oxygen values were found in the bottom layer at the shelf (<0.5 mL·L^−1^) and shelf break (<1.0 mL·L^−1^) stations below 100 m and 150 m, respectively. At the first glance, it seems that the low oxygen concentrations have an effect on the vertical distribution of the Chaetognatha because of the high number of Chaetognatha found above these low oxygen layers. However, the standing stock was mainly composed of species belonging to the Serratodentata group (61.3% at the inner shelf station, 89.7% at the outer shelf station, 36.2% at the shelf break station), which is characterized as an epipelagic group [41] having a WMD between 34 m and 105 m. Other species, such as *E. hamata*, *S. decipiens, S. hexaptera* and *S. lyra*, were found in these low dissolved oxygen layers (Figure 2 and 5). This showed that these species were not excluded by low oxygen concentrations. Auel and Verheye [42] state that abundant copepod species in this area such as *Metridia lucens*, *Paraeucalanus robusta*, *Heterorhabdus* sp., *Aetidopsis carrinata* and *Rhincalanus nasutus* are able to tolerate low oxygen levels. Moreover, they can be very abundant in these layers. Therefore, it seems likely that the vertical distribution of Chaetognatha is more driven by the prey abundances than by environmental factors.

Within the vertical distribution of Chaetognatha species a segregation of maturity stages on the Walvis Bay transect was not clearly detected. Kehayias *et al.* [41] determined that epipelagic species do not exhibit ontogenetic vertical distribution, which could be caused by the relatively small depth range, characterizing these species (0–200 m). The community was dominated by epipelagic to shallow-mesopelagic species. Seven epipelagic and three epipelagic to shallow-mesopelagic species were found on the Walvis Bay transect (Table 5). Some adults of these species such as *P. draco* or *S. decipiens* occurred in deeper layers than the juveniles (Figure 6 and Table 6). However, these differences were not evident enough to make a clear statement about the ontogenetic distribution. It was difficult to determine the ontogenetic vertical distribution of the deep-mesopelagic and bathypelagic species because the community of these species was dominated by juveniles which may be associated with the winter spawning [37]. Gibbons and Verheye [43] assume that Chaetognatha stage structure coupled with an ontogenetic distribution can influence the vertical distribution of the total population. This may have affected the classification of the species as deep-mesopelagic or bathypelagic in this study.

The richness and diversity of Chaetognatha species along the Walvis Bay transect increased from the shelf towards the open ocean (Table 7). The most obvious reason for these findings is the water depth of the area. At the inner and outer shelf stations, the water depth was 132 m and 231 m, respectively. These two stations were dominated by epipelagic species such as *S.tasmanica, S. decipiens, S. sibogae* and *S. minima,* but some mesopelagic species were found (*E. hamata, S. hexaptera*) as well. At the shelf break station, one deep-mesopelagic species was detected (*S. zetesiois*). At the offshore station, all categories were represented. Another reason for the increase of richness and diversity from the shelf towards the open ocean is the occurrence of oceanic species such as *S. minima, S. lyra* and *P. draco* at the shelf break and offshore stations.

The Serratodentata group dominated the Chaetognatha community at the shelf and shelf break stations (D >0.31). The high abundances of the species belonging to this group are in concordance with a study by Duró and Gili [17]. Resulting from the high abundance of the Serratodentata group at the shelf stations and the high diversity at the shelf break and offshore stations, higher variations in community evenness between species were found on the shelf (J <0.33).

### Stable Isotopes

Nitrogen isotopic composition reflects important dietary relationships within the food web [29]. The trophic position of Chaetognatha could be determined using an enrichment factor of 3.4 ‰ per trophic level [44]. Phytoplankton δ^15^N values were not available for this area. Hence, seston values were used as baseline. The values ranged between 3.29 ‰ and 4.73 ‰ with 4.73 ‰ at the inner shelf station, 3.83 ‰ at the outer shelf station, 3.29 ‰ at the shelf break station, and 4.19 ‰ at the offshore station [45]. The lowest δ^15^N values were found near the surface in upper 30 m thus reflecting a higher productivity [46].

In addition, Koppelmann *et al*. [45] examined the δ^15^N and δ^13^C values of thecosomatic Pteropoda in the Northern Benguela. Thecosomata are considered to be predominantly herbivorous, capturing food with a mucous web [47]. Koppelmann *et al*. [45] confirmed that Thecosomata occupy a trophic level between primary producers (phytoplankton) and carnivorous organisms like Chaetognatha in the food web. The Chaetognatha δ^15^N values (8.96±1.19‰ at the offshore station, 8.33±1.62 ‰ at the shelf break station, 9.28±0.86 ‰ at the shelf station) were 3–4 ‰ higher than those of Thecosomata (4.56±0.45 ‰ at the offshore station, 5.50±0.70 ‰ at the shelf break station, 5.39±0.50 ‰ at the shelf station). This supports the expected theory that Chaetognatha are both secondary and tertiary consumers.

The lowest δ^13^C values were found at the Rocky Point offshore station which statistically differs from all other areas. These differences could be caused by different water masses at the Rocky Point offshore station which was the farthest station from the coast. Several factors may contribute to the variability of δ^13^C, e.g., temperature differences, composition of the phytoplankton as well as differences in the growth rates of phytoplankton.

The stable isotope ratios of four taxa (*S. minima,* Planctonis group, *S. enflata, S. decipiens*) were determined. The δ^15^N values from *S. decipiens* were higher (9.16 ‰ to 10.05 ‰) than those found in other species. The lowest δ^15^N values were found for *S. minima.* These differences could be a result of the body size of the species and hence of a different food composition, i.e., body size correlates with prey size. *S. minima* is the smallest species found on the Walvis Bay transect with a maximal body length of 7–10 mm [2]. The potential prey of this species in the Northern Benguela are Copepoda, Chaetognatha and Crustacea larvae [43]. The small body size of *S.minima* suggests that this species feed on smaller Copepoda species. Contrasting to *S.minima*, *S. enflata* and *S. decipiens* reach a body length up to 25 mm [48] and 14 mm [2], respectively. These larger species have a wider range of potential prey items. As the *S. decipiens, S. enflata* are animals of higher trophic level, the nitrogen isotope content of these species may reach higher values. The prey composition of *S. zetesiois* and *S.planctonis* has not been closely investigated yet.

As discussed above, the results of the stable isotope analyses varied between the areas. For the determination of the stable isotopes of the four taxa, samples taken on different stations were pooled. However, the values of the four taxa did not differ greatly. No significant differences were found between the areas where the samples for the determination of stable isotopes of the taxa were taken.

### Body Composition

Body composition can vary with season [49] and locations [50]. The body composition is possibly influenced by dissimilar life cycles and nutritional conditions of animals [51]. Ikeda [51] found that the nitrogen content of zooplankton changes greatly from species to species and no consistent relation between body size and habitat temperature exists. In this study, differences between the relative nitrogen content of the species were found. The relative nitrogen values of *S.enflata* (from 8.77±1.08% to 9.93±0.78%) are similar to those found by Gorsky *et al.* [52], (9.1±2.8% in 1988) and Batistić [53], (9.58±2.04% in 2003). The relative nitrogen amount of *S. minima* was 7.14±1.55%. Batistić [53] and Gorsky *et al.* [52] reported higher values of 11.88±0.70% and 11.80±0.20%, respectively. Differences in the nitrogen content may be caused by differences in the life history and in seasonal trends of zooplankton production at different localities [54].

The results of the relative carbon content of Chaetognatha species determined in this study are generally similar to those found in other studies [52]–[53]. However, in detail there are some differences. In this study, the relative carbon content of *S. enflata* varied from 40.70±8.91% to 49.94±2.96% at the stations. The percentage of carbon of *S. enflata* determined by Batistić [53] ranged between 30.41±2.53% and 40.39±1.63%. Gorsky *et al.* [52] reported carbon percentage of 43.7±11%. The relative carbon content of S. *minima* determined in this study (31.98±9.58%) is similar to values published by Batistić [53] 27.73±2.68% and 39.31±1.99%. For *S. minima,* Gorsky *et al*. [52] reported higher relative carbon values (51.0±2.1%). The higher relative C values investigated for *S. minima* and *S. enflata* in this study may originate in the different developmental stage composition of Chaetognatha in the samples. Carbon and nitrogen content of some species depends on the maturity stage and increases with age, thus with dry weight and length class [53]. Gorsky *et al.* [52] sampled during a spring-season in the north-western Mediterranean Sea. In this period more mature individuals occurred in higher numbers [41]. Another possibility for these variations may be differences in the area and time of sampling. In the north-western Mediterranean Sea, temperatures can reach high values up to 25°C during spring [55]. Batistić [53] sampled in the Adriatic Sea at different times of the year and temperatures ranged from 12°C in February to 22.5°C in August. The temperature measured in spring in the BUS was lower than 16°C.

The high carbon values reflect a high proportion of organic matter in the animal body and low nitrogen values indicate low proportions of protein. The C:N ratio is a useful index to distinguish protein from fat. The ratio varied greatly from 4.5 for some Copepoda species to 45 for Leptomedusae [50]. The C:N ratio of different Chaetognatha species described in this study ranged between 5.25±0.42 and 6.20±0.36. Kruse *et al.* [56] observed that C:N ratios found for species belonging to Eukrohniidae could vary between 4.3±0.6 and 5.1±1.0. Also, the C:N ratios determined by Gorsky *et al.* [52] were lower and varied between 4.0±0.7 and 5.0±1.4. The higher C:N ratio of Chaetognatha occurring in the BUS suggested that larger amounts of fat were saved in the animal body [50]. The high lipid content may help the animal to survive periods of poor food concentrations which are typical for pulsed upwelling systems [18]. The C:N ratios of Chaetognatha determined in this study were close to the C:N ratio of zooplankton determined by Redfield (6.24) [57].

### Conclusion

In this study, differences in the horizontal distribution of the Chaetognatha species along the Walvis Bay transect were observed. The community of Chaetognatha was dominated by epipelagic and shallow-mesopelagic species. No effect of low oxygen concentrations on the vertical distribution of Chaetognatha was detected. Therefore, we assumed that the vertical distribution of Chaetognatha species is more driven by the prey abundances than by environmental parameters.

Findings in this study support the view that the Chaetognatha of the Benguela Upwelling System (BUS) play a significant role as secondary and tertiary consumers in the food web. This study will help understand the trophic relationships between the different taxonomical groups in the BUS and will contribute to the calculation of material fluxes within the pelagic food web.
